# Vapour Application of Sage Essential Oil Maintain Tomato Fruit Quality in Breaker and Red Ripening Stages

**DOI:** 10.3390/plants10122645

**Published:** 2021-12-01

**Authors:** Antonios Chrysargyris, Charalampos Rousos, Panayiota Xylia, Nikolaos Tzortzakis

**Affiliations:** 1 Department of Agricultural Sciences, Biotechnology and Food Science, Cyprus University of Technology, Limassol 3036, Cyprus; a.chrysargyris@cut.ac.cy (A.C.); rousos9@hotmail.com (C.R.); pa.xylia@edu.cut.ac.cy (P.X.); 2 Department of Life Sciences, School of Sciences, European University of Cyprus, Nicosia 1516, Cyprus

**Keywords:** fruit storage, natural products, quality-related attributes, tomato, volatiles

## Abstract

Consumers seek safe, high-nutritional-value products, and therefore maintaining fresh produce quality is a fundamental goal in the food industry. In an effort to eliminate chemical-based sanitizing agents, there has been a shift in recent decades toward the usage of eco-friendly, natural solutions (e.g., essential oils-EOs). In the present study, tomato fruits (*Solanum lycopersicum* L. cv. Dafni) at breaker and red ripening stage were exposed to sage essential oils (EO: 50 μL L^−1^ or 500 μL L^−1^) for 2, 7 and 14 days, at 11 °C and 90% relative humidity (RH). Quality-related attributes were examined during (sustain effect—SE) and following (vapour-induced memory effect—ME; seven days vapours + seven days storage) vapour treatment. In breaker tomatoes, EO-enrichment (sustained effect) retained fruit firmness, respiration rates, and ethylene emission in low EO levels (50 μL L^−1^). In contrast, breaker fruit metabolism sped up in high EO levels of 500 μL L^−1^, with decreased firmness, increased rates of respiration and ethylene, and effects on antioxidant metabolism. The effects were more pronounced during the storage period of 14 days, comparing to the fruit exposed to common storage-transit practice. In red fruits, the EOs impacts were evidenced earlier (at two and seven days of storage) with increased rates of respiration and ethylene, increased *β*-carotene, and decreased lycopene content. In both breaker and red ripening fruit, EO application decreased weight losses. Considering the fruits pre-exposed to EOs, quality attributes were more affected in green fruits and affected to a lesser level in the red ones. Furthermore, based on appearance, color, and texture evaluations, organoleptic trials demonstrated an overwhelming preference for EO-treated red fruit during choice tests. EOs had lower effects on total phenolics, acidity, total soluble solids, and fruit chroma, with no specific trend for both breaker and red tomatoes. Natural volatiles may aid to retain fruit quality in parallel with their antimicrobial protection offered during storage and transportation of fresh produce. These effects may persist after the EO is removed from the storage conditions.

## 1. Introduction

The increased demands on fresh produce, fruits, vegetables, and herbs is challenged nowadays, with efforts focusing on the increasing yields and quality during the crop production. Moreover, efforts have also been targeted to decrease produce losses during the postharvest storage. As a consequence, the increased consumption of fresh produce has driven commercial desire for better storage and transportation conditions. There is an increased interest on effective sanitation means a decrease in postharvest losses due to decay, while maintaining fruit quality, including flavor, color, nutritional value, texture, and storability [1,2]. Non-single preservation means are efficient enough to be applied in a wide range of fresh produce, microorganisms, and environmental conditions, for each crop. Despite the fact that chemical applications in postharvest are of high effectiveness, there are significant challenges including current sanitation procedures and health and environmental concerns due to the possible generation of toxic by-products and residues [3,4,5]. Moreover, the use of chemicals as fungicides for postharvest sanitation is partially restricted in many countries [6]. Therefore, alternative, safe, eco-friendly but effective sanitizing agents are explored for the fresh produce preservation [7,8,9,10,11].

One candidate is the essential oils (EOs) derived from medicinal and aromatic plants (MAP) due to a wide range of biocidal activities, including antifungal [12,13,14], antibacterial [15,16,17], antioxidant [15,17], cytotoxic [18], and anti-inflammatory [19] activities, to name a few. Essential oils from a variety of plant species, including sage (*Salvia* spp.), have demonstrated bioactivity against a variety of plant diseases [7,20,21]. However, there has been not much research on the beneficial effects of the EOs application on the fruit quality of pears, tomatoes, eggplants, strawberries, and cherries [2,6,12,22,23], processed fresh produce [24] and cut flowers [25,26]. Although the strong aroma of EO can restrict the final product’s organoleptic acceptability, it is known to have strong antioxidant and antimicrobial properties [7,27,28]. Recent research has revealed that EO (i.e., *Thymus capitatus*; thyme oil), can act as signaling compound. Therefore, EO application is triggering a signal to induce a defense mechanism in vegetables by increasing the activity of defense-related enzymes and increasing antioxidant ability [29]. Essentials oils can be both applied in aqueous and in vaporized phase, with the latter being an advantage for some commodities (i.e., strawberries and grapes) where aqueous sanitation is a limitation. The EO’s antimicrobial activity is linked to its hydrophobic properties, which allow it to penetrate into microbial cells’ phospholipid membranes, causing structural disorder and permeability [30]. However, the use of EOs in high levels is restricted due to probable unfavorable sensory effects, and as a result of that, the concentrations used need to be optimized for each commodity.

Tomatoes (*Solanum lycopersicum* Mill.) are harvested at different stages of ripeness to meet various consumption needs (e.g., fresh and processed). For red-fleshed tomatoes, six ripeness stages (i.e., green, breaker, turning, pink, light red, and red) are described based on the surface color [31]. Breaker-turning ripeness stage is used for longer fruit storage and transportation. Tomato is a climacteric fruit with a limited postharvest life due to the elevated levels of respiration, transpiration, ethylene emission and postharvest decay, resulting in an increased ripening process and senescence [32,33]. Tomato ripening is accompanied with chlorophyll break down, lycopene accumulation, tissue softening, and shifts in aroma and other compositional properties [34]. Following harvest, the fruit continues to have several biochemical changes on quality and deteriorates rapidly. In some cases, fruit deterioration can be during or after transport and marketing. Tomatoes are stored at comparably high temperatures (10–12.5 °C) depending on the maturity stage to prevent chilling injury which is evidenced at lower temperatures, below 7–10 °C [35].

Only fresh produce that meets the consumer’s standards is suitable at the market interface. As a result, it is vital to assess the impact of potentially revolutionary applications on the sensory and organoleptic features of fruits and vegetables. Sage EO has been effective in fruit quality and observed antimicrobial activity [23,36]. The goal of this study was to examine if the vapor phase of essential oils obtained from sage (*Salvia trilova* L.) had any effect on tomato fruit quality attributes including: (i) physiological parameters (including weight loss, fruit firmness, and rates of respiration and ethylene production); (ii) fruit chemical composition (for example, vitamin C content, antioxidant capability, organic acid content (citrate), total soluble solids, carotenoids (lycopene, *β*-carotene) and total phenolic content) and damage index; and (iii) sensory qualities as determined by a consumer panel under controlled settings.

## 2. Results

**Figure 1:**
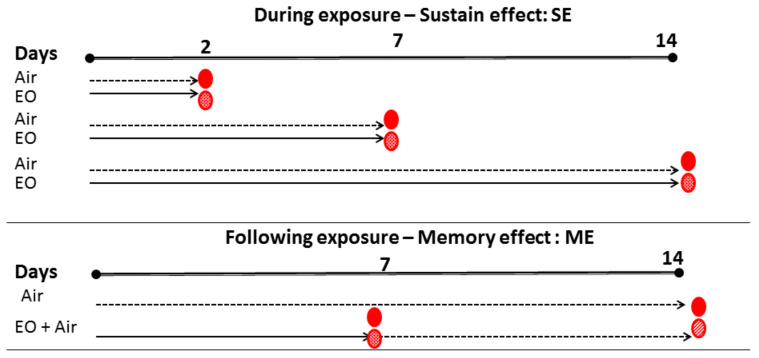
Layout of experiments: Tomato fruit were exposed to ambient air or essential oil (EO: 50 or 500 μL L^−1^) in the dark at 11 °C and 90% RH. Experiment 1: Tomato fruit were exposed to air or EO for 2, 7, and 14 days and sampling took place during exposure (sustain effect—SE) to air or EO. Experiment 2: Tomato fruit were exposed to air or EO for seven days, and then transferred for additional seven days to air. Sampling took place following EO exposure (memory effect—ME) at 14 days of storage. Air (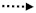), EO exposure (→). Tomato exposed to air 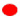, tomato exposed to EOs 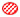.

The experimental lay out is presented in Figure 1, with tomato fruits exposed to sage EO (50 or 500 μL L^−1^) during storage for up to 2, 7, and 14 days or exposed to EO for 7 days and then stored to chilled conditions for an additional 7 days.

### 2.1. Fruit Decay

Neither the EO-treated nor the control fruit showed signs of degradation until day 7 of the storage period. At the end of the trial (day 14), control fruit showed evidence of deterioration (assessed as 2.05 and 2.75 on a 1–5 scale, for breaker and red fruit, respectively) [principally symptoms of anthracnose rot (caused by *Colletotrichum coccodes*) and secondary symptoms of black spot] (caused by *Alternaria alternata*)] as shown in Table 1.

### 2.2. Fruit Weight Loss, Firmness and Colour

**Figure 2:**
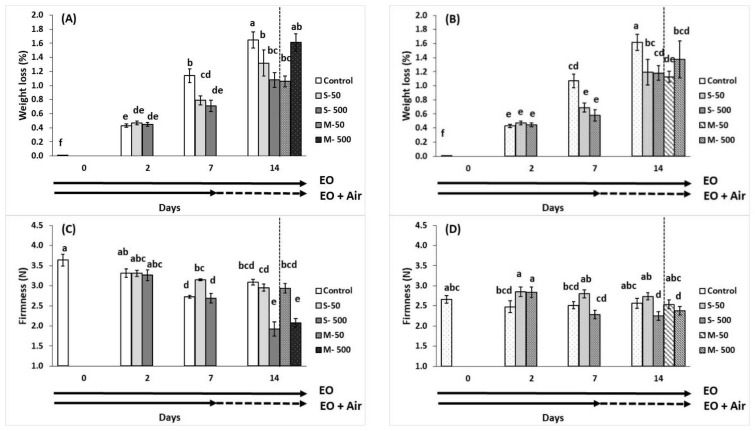
Impacts of sage essential oil (EO) on weight loss (%) and firmness (expressed in Newtons) of tomato fruit at breaker (**A**,**C**) and red (**B**,**D**) ripening stage, exposed to ambient air (control) or EO (50 or 500 μL L^−1^) for 2, 7, and 14 days (sustain effect—S) or up to 7 days and then transferred to ambient air for an additional 7 days (memory effect—M). Fruits were maintained throughout at 11 °C and 90% RH. Values represent mean (±SE) of measurements made on eight independent fruit per treatment and storage period. Means followed by different Latin letters significantly differ according to Duncan’s MRT (*p* = 0.05).

Fruit weight loss increased when storage time was extended, reaching 1.65% in control and in 1.32% in 500 μL L^−1^ EO-treated breaker fruit after 14 days at 11 °C (Figure 2A) while the relevant values in red fruits were 1.61% and 1.17%, respectively (Figure 2B). Fruit weight loss (%) was at similar levels for both breaker and red tomatoes during 2 days of storage. However, fruit weight loss was significantly decreased (up to 45%) in EO-treated tomatoes after 7 and 14 days, comparing with fruits maintained throughout in ambient air at 11 °C (Figure 2A,B). Interestingly, the effects were persisted when fruit removed from 50 μL L^−1^ of EO exposure (including 500 μL L^−1^ of EO for red tomatoes), and stored additionally for seven days (memory effect).

The effect of EO and fruit ripening stage on the tomato firmness is presented in Figure 2C,D. Two days’ storage in an EO-enriched atmosphere revealed no changes in the tomato firmness for breaker stage fruits, but maintained in red tomatoes. Tomatoes-enriched with 50 μL L^−1^ EOs maintained fruit firmness up to 14 days comparing with higher concentration (500 μL L^−1^) in both red and breaker fruits. However, when treated fruit was transferred to ambient air, breaker and red fruit previously exposed to 50 μL L^−1^ EO remained substantially (*p* = 0.01) firmer than fruit subjected to 500 mg L^−1^ EO storage conditions throughout.

Fruit colour was mainly affected by the storage and ripening stage of tomatoes rather than the EO application (Figure S1). At breaker stage, L* value was greater in 500 μL L^−1^ EO application at 14 days of storage for both sustain and memory treatments (Figure S1A). Moreover, breaker-tomatoes revealed decreased chroma and a* value but increased b* value in 500 μL L^−1^ EO application at 14 days of storage (Figure S1C,G). Red tomatoes revealed increased L* value at 2 and 7 days but decreased Chroma and a* value at high EO (500 μL L^−1^) application at 14 days of storage (Figure S1B,D,H).

### 2.3. Soluble Solids, Organic Acid and Ripening Index

**Figure 3:**
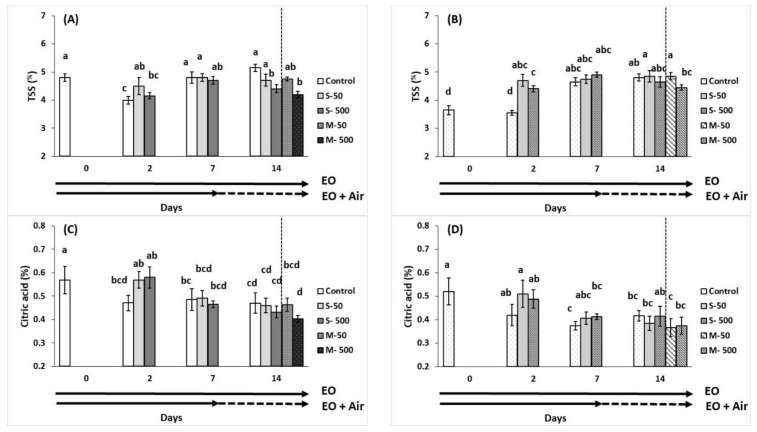
Impacts of sage essential oil (EO) on total soluble solids (TSS; in %) and acidity (% citric acid) of tomato fruit at breaker (**A**,**C**) and red (**B**,**D**) ripening stage, exposed to ambient air (control) or EO (50 or 500 μL L^−1^) for 2, 7, and 14 days (sustain effect—S) or up to 7 days and then transferred to ambient air for an additional 7 days (memory effect—M). Fruits were maintained throughout at 11 °C and 90% RH. Values represent mean (±SE) of measurements made on eight independent fruit per treatment and storage period. Means followed by different Latin letters significantly differ according to Duncan’s MRT (*p* = 0.05).

Total soluble solids (TSS) levels attained a maximum after 14 days of storage for breaker and red tomatoes (Figure 3A,B). Two days’ storage in an EO-enriched atmosphere revealed in increased levels for TSS content in red tomatoes. However, soluble sugar levels were found reduced in breaker tomatoes either exposed to EO (500 μL L^−1^) for 14 days or pre-exposed to EO for 7 days and then transferred to ambient air (Figure 3A). Citric acid content measured by titratable acidity (TA), was declined (*p* < 0.05) as fruit ripened (Figure 3C,D), whereas EO application in general resulted in no changes in the citric acid content. Ripening index indicated by the ratio of TSS/TA did not differ among the tested applications and/or storage period (data not presented).

### 2.4. Respiration Rate and Ethylene Emission

**Figure 4:**
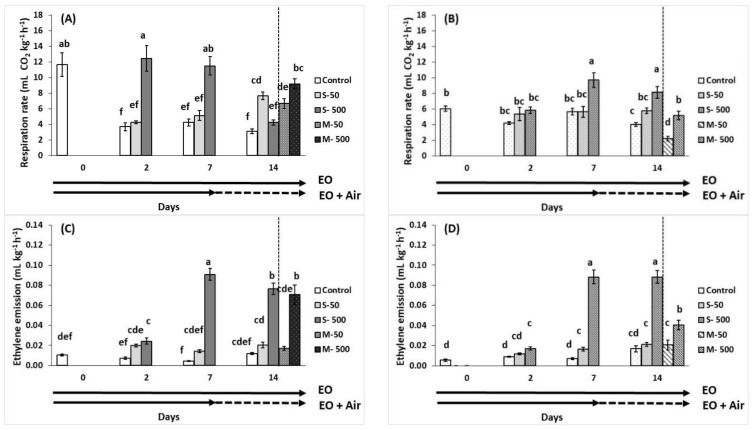
Impacts of sage essential oil (EO) on respiration rate (mL CO_2_ kg^−1^ h^−1^) and ethylene emission (mL kg^−1^ h^−1^) of tomato fruit at breaker (**A**,**C**) and red (**B**,**D**) ripening stage, exposed to ambient air (control) or EO (50 or 500 μL L^−1^) for 2, 7, and 14 days (sustain effect—S) or up to 7 days and then transferred to ambient air for an additional 7 days (memory effect—M). Fruits were maintained throughout at 11 °C and 90% RH. Values represent mean (±SE) of measurements made on eight independent fruit per treatment and storage period. Means followed by different Latin letters significantly differ according to Duncan’s MRT (*p* = 0.05).

Fruit treated with volatiles (500 μL L^−1^) revealed an increased respiration rate after 2 and 7 days at breaker tomatoes and after 7 and 14 days of storage at red tomatoes (Figure 4A,B). Low level (50 μL L^−1^) EO-treated tomatoes at breaker stage respired greater than the relevant control (fruits stored in ambient air) following 14 days of storage. Respiration rate was increased in pre-exposed fruit to EO (500 μL L^−1^) for red and for breaker (including the 50 μL L^−1^ EO) tomatoes. Indeed, red tomatoes pre-exposed to 50 μL L^−1^ EO and followed 7 days of storage revealed the lowest respiration rate (Figure 4B).

Similar trend to respiration rates was observed in fruit ethylene production (Figure 4C,D). Therefore, the high EO (500 μL L^−1^) concentration increased the ethylene emission in both exposed and pre-exposed tomatoes to EO, for both breaker and red maturation tomato stages.

### 2.5. Carotenoid Composition and Ascorbic Acid

**Figure 5:**
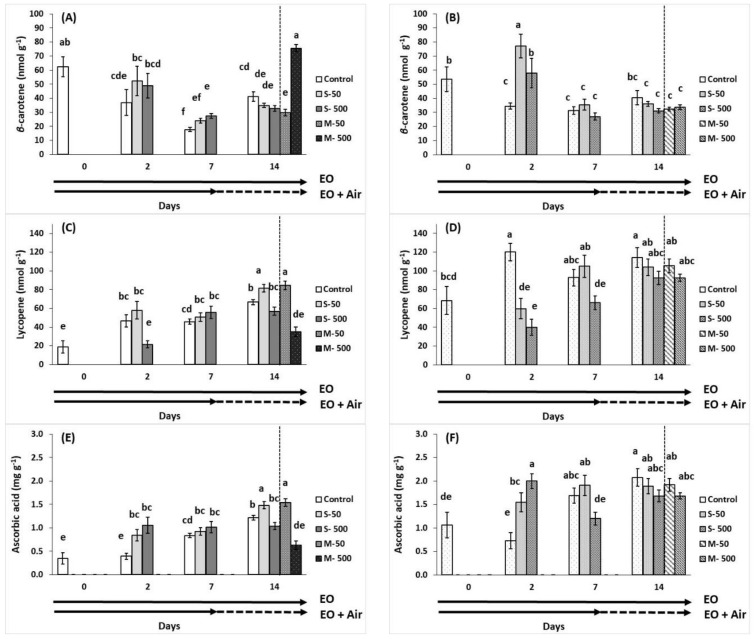
Impacts of sage essential oil (EO) on *β*-carotene (nmol g^−1^), lycopene (nmol g^−1^) and ascorbic acid (mg g^−1^) in tomato fruit at breaker (**A**,**C**,**E**) and red (**B**,**D**,**F**) ripening stage, exposed to ambient air (control) or EO (50 or 500 μL L^−1^) for 2, 7, and 14 days (sustain effect—S) or up to 7 days and then transferred to ambient air for an additional 7 days (memory effect—M). Fruits were maintained throughout at 11 °C and 90% RH. Values represent mean (±SE) of measurements made on eight independent fruit per treatment and storage period. Means followed by different Latin letters significantly differ according to Duncan’s MRT (*p* = 0.05).

In breaker fruits, EO application at 500 μL L^−1^ increased *β*-carotene content at 7 days of storage but this effect did not persist after 14 days of storage (Figure 5A). Fruits pre-exposed to 500 μL L^−1^ of EO and stored for additional seven days in ambient air marked an increase in *β*-carotene content, almost twice more than the control fruits. A steady increase in *β*-carotene content marked at two days of volatiles application in red tomatoes (Figure 5B).

Sage oil-treated fruit at breaker and red ripening stage with 50 μL L^−1^ maintained or increased lycopene content at 7 and 14 days of exposure, while lycopene content in red tomatoes reduced due to EO application at 2 days of storage (Figure 5C,D). Lycopene content was significantly (*p* < 0.01) reduced in pre-exposed breaker tomatoes to 500 μL L^−1^ EO, following storage of seven days, but such effects were not found in the relevant red tomatoes, tomatoes pre-exposed to 500 μL L^−1^ EO, and storage of seven days.

EO-enrichment resulted in increased ascorbic acid (AA) content in breaker fruits at 2 days and this effect was persisted in 50 μL L^−1^ EO-treated fruits at 14 days and in the pre-exposed fruits with 50 μL L^−1^ EO and stored for 7 days in ambient air (Figure 5E). Indeed, the relevant pre-exposed fruits with 500 μL L^−1^ EO had decreased AA content compared with the control at 14 days of storage. The non-treated fruits with EOs, revealed increased levels of AA during the storage period. Similarly, in red tomatoes, EO-treated fruits had increased AA content at 2 days but this effect was not persisted thereafter (Figure 5F).

### 2.6. Total Phenols Content and Antioxidant Activity

**Figure 6:**
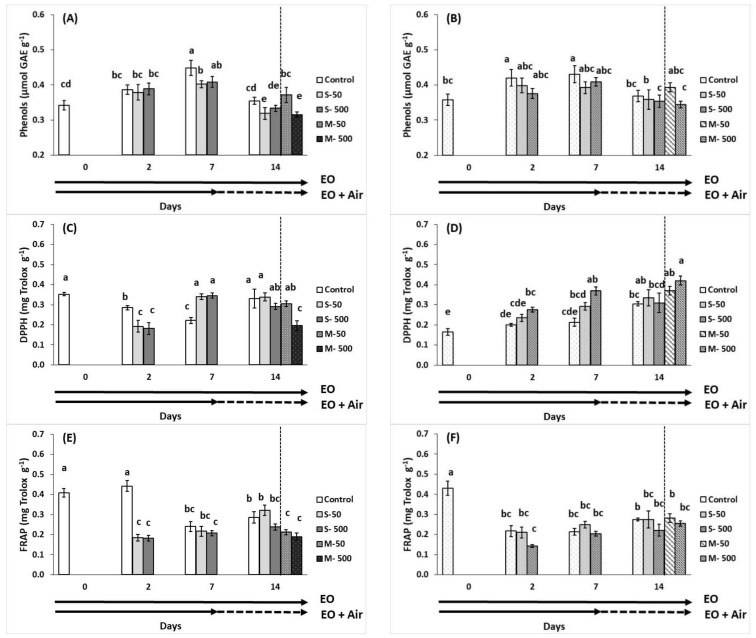
Impacts of sage essential oil (EO) on total phenolics (GAE μmol g^−1^) and antioxidant activity (mg Trolox g^−1^) in tomato fruit at breaker (**A**,**C**,**E**) and red (**B**,**D**,**F**) ripening stage, exposed to ambient air (control) or EO (50 or 500 μL L^−1^) for 2, 7, and 14 days (sustain effect—S) or up to 7 days and then transferred to ambient air for an additional 7 days (memory effect—M). Fruit were maintained throughout at 11 °C and 90% RH. Values represent mean (±SE) of measurements made on eight independent fruit per treatment and storage period. Means followed by different Latin letters significantly differ according to Duncan’s MRT (*p* = 0.05).

EO-treatment tended to decrease or not to affect fruit total phenols content, and the effects did not attain statistical significance (Figure 6A,B). However, non-treated fruits with EOs, revealed increased levels of total phenolics up to seven days of storage, but this effect did not persist thereafter. Antioxidant activity measured by ferric-reducing antioxidant power (FRAP) and 2,2-diphenyl-1-picrylhydrazyl (DPPH) radical scavenging activity methods was reduced in breaker tomatoes exposed to EO for two days. However, at seven days of storage DPPH activity was increased in EO-treated breaker tomatoes (Figure 6C,E). Moreover, pre-exposed fruit to 500 μL L^−1^ of EO following storage of additional 7 days in ambient air revealed decreased DPPH levels, compared to the relevant control at 14 days of storage. In red tomatoes, DPPH antioxidant capacity of fruit was increased with the 500 μL L^−1^ of EO application, and this effect was persisted in pre-exposed fruits as well (Figure 6D). FRAP activity was not changed during storage and/or EO application (Figure 6F).

### 2.7. Plant Stress Indicators

**Figure 7:**
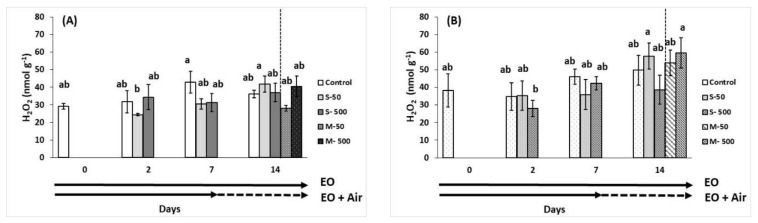

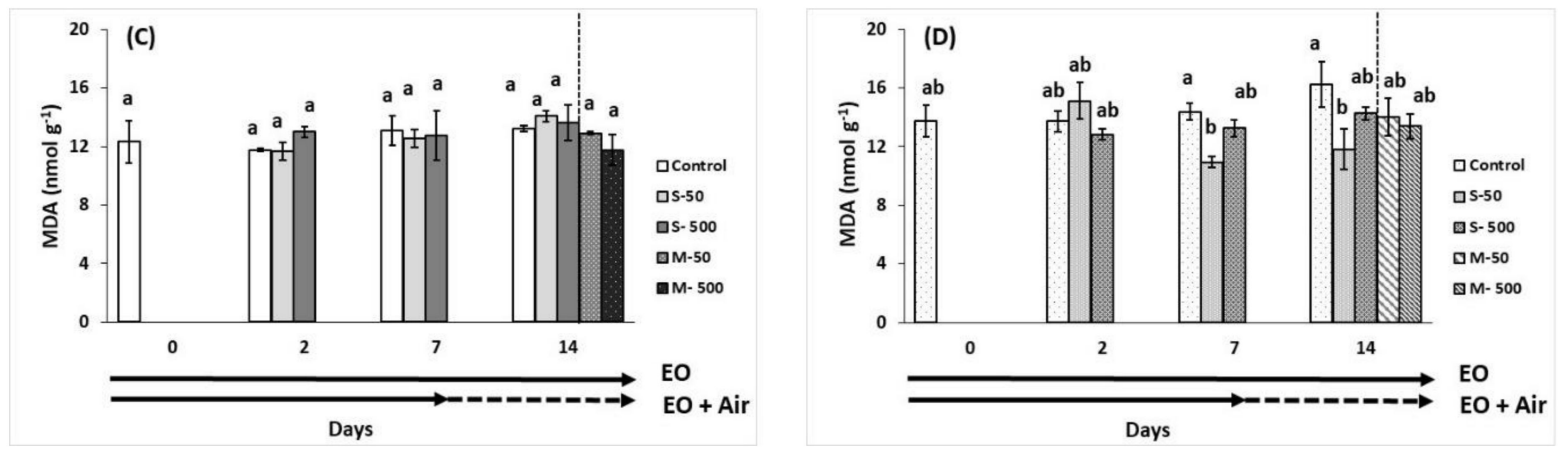
Impacts of sage essential oil (EO) on the fruit content of hydrogen peroxide (H_2_O_2_) and malondialdehyde (MDA) in tomato fruit at breaker (**A**,**C**) and red (**B**,**D**) ripening stage, exposed to ambient air (control) or EO (50 or 500 μL L^−1^) for 2, 7, and 14 days (sustain effect—S) or up to 7 days and then transferred to ambient air for an additional 7 days (memory effect—M). Fruits were maintained throughout at 11 °C and 90% RH. Values represent mean (±SE) of measurements made on eight independent fruit per treatment and storage period. Means followed by different Latin letters significantly differ according to Duncan’s MRT (*p* = 0.05).

In breaker and red fruits, hydrogen peroxide (H_2_O_2_) fluctuated among the treatments without a specific trend (Figure 7A,B). In breaker stage fruits, lipid peroxidation as assessed in terms of malondialdehyde (MDA) content, was not changed in EO-treated fruits and/or during storage duration of 14 days (Figure 7C). However, in red tomatoes MDA decreased at 50 μL L^−1^ of EO application after 7 and 14 days of storage (Figure 7D).

### 2.8. Sensory Evaluation

Sensory evaluation revealed significant changes between treatments that the jurors were able to detect and marked as 86% and 50% for breaker and red tomatoes. In breaker tomatoes, jurors preferred un-treated fruit with 57% and followed by 50 μL L^−1^ treated fruits with 43%. In the case of the red tomatoes, jurors preferred low EO-treated fruits and then control fruits with 79% and 21%, respectively. Interestingly, no preference was noticed for high-EO treated tomatoes, revealing the lowest sensory scores (Table 2). Those who preferred low EO-treated fruits did so due to the improved appearance and texture, which was comparable to that of the untreated fruits (Table 2). In red tomatoes, taste panelists preferred fruit subjected to low-level EO-enrichment (50 μL L^−1^), and this effect was mirrored by the increased scores in appearance, color, aroma, texture, and sweetness.

## 3. Discussion

Only fresh produce that meets the consumer’s standards is suitable at the market interface. As a result, evaluating the impact of possible novel procedures on the sensory and organoleptic features of fruits and vegetables is critical. Weight loss, color, firmness, total soluble solids, total acidity, and antioxidants are only a few attributes that affect postharvest fruit quality. Moreover, the postharvest performance of the tomato ripening stage and understanding the physiological changes taken place during storage are of high research interest [37]. In the present study, mature green tomato fruit when were subjected to EO-enrichment (sustained effect) were perceptibly retained their firmness in low EO levels (50 μL L^−1^). However, the rates of respiration and ethylene as well as the antioxidant metabolism were increased in high EO levels of 500 μL L^−1^, and the effects were more pronounced during the storage period of 14 days, in comparison to the control fruits (subjected to typical storage and transportation methods). When red tomatoes (being in higher maturation stage compared to mature green fruit) were subjected to EOs, effects on quality attributes were appeared even earlier, after two days of EOs exposure, with increase of TSS, and *β*-carotene and decrease on lycopene content. Considering the pre-exposed fruits to EOs, quality attributes were more affected in mature green fruits and to a lesser level in the red fruits. Furthermore, based on appearance, color, and texture evaluations, taste panel trials demonstrated an overwhelming preference for EO-treated red fruits during choice testing.

The relationship between increased ethylene production and tomato ripening is well understood [33] and effects are related to the fruit ripening stage, by altering signaling genes related to ethylene metabolic pathway [37]. In addition to the ripening stage, biotic and abiotic stresses have an impact on ethylene production [38]. In tomato fruit, the increase in respiration occurs either concurrently or shortly after the increase in ethylene production [39,40] and this was evidenced in both mature-green and red tomatoes, starting from the 2nd day up to 14th day of storage for the high EO concentration of 500 μL L^−1^. Interestingly, mature green tomatoes stimulated more the respiration rates compared to the relevant red tomatoes with the EO of 500 μL L^−1^. When ethylene is added to mature-green tomato fruit, ethylene hastens the climacteric and ripening process [37], meaning induced respiratory climacteric due to increased endogenous ethylene output in tomato [41]. That could be the case in our study, but further research is needed to that direction before final conclusions. As a result, a comprehensive investigation at the molecular level is required to investigate the effects of EOs on gene and/or protein expression in metabolic pathways, such as the ethylene biosynthesis pathway, which is linked to fruit ripening (particularly in climacteric fruits such as tomatoes).

Fresh commodities loss weight mainly by vapor pressure at different locations [42] but also through the respiration process [43]. A loss of more than 5%, on the other hand, is a limiting factor for the fruit marketing and consumption [44]. However, weight loss in the present study was <1.8%. Fruit treated with EO lost less weight during storage than fruit that had not been treated with EOs, and weight loss increased progressively over time. This decrease in weight loss could be attributed to the ability of the EOs to decrease water exchange and solute movement due to EOs hydrophobic properties [45]. The ability of the essential oil to act as a barrier and the antioxidant activity of the essential oil coatings were responsible for the reduced weight loss rate in coated fruits during storage [46,47].

During fruit maturation and storage time, titratable acidity is decreased and TSS is increased in general, as this trend was observed in our study. TA was decreased up to 17% from day 0 to day 14, and the values were ranged from 0.2 to 0.6%, being in agreement with previous records [40].

EO application as preservative means is well documented due to their antimicrobial and antioxidant activities [12,13,48,49]. Moreover, sage (*Salvia officinalis* L.) antibacterial and antifungal properties have been reported previously [50]. In the present study, sage EO maintained their antimicrobial efficiency up to 14 days, with reduced decay symptoms, being in accordance with previous applications of cinnamon and eucalyptus EO on tomatoes and strawberries [22]. In the present study, the main component of sage EO was eucalyptol, as described at the Section 4, with proven antimicrobial activity [51,52]. Additionally, secondary components of sage EO, such as camphor and *α*-pinene have also antimicrobial activity [53,54]. Both primary and secondary components of an EO contribute to the antimicrobial activity of the oil, affecting the quality of the fresh produce. Fruit decay causes metabolic alterations that are responsible for unpleasant smell and flavor [40]. Based on the findings of this study, it is hypothesized that the active component in sage EO continues to be released throughout storage, extending the fruit’s shelf-life. Additionally, the effects were persisted even when fruits removed from the EO and were stored in ambient air for seven days, indicating a residual effect. Similarly, sage EO revealed residual effects in pepper fruits [36].

The pigment content of the fruit changes during development, whereas the chlorophyll level falls during ripening, prompting the synthesis of carotenoids, including the red pigment lycopene as well as *β*-carotene. In red tomatoes 500 μL L^−1^ EO-treated fruits had lighter (higher L* value) color than the untreated ones during two and seven days of storage at 11 °C, suggesting delayed color development by EO treatment [55]. This was evidenced by the decreased lycopene levels for the EO-treated fruits up to seven days of storage. However, this effect did not persist after 14 days of storage. Noticeably, delay in color development was evidenced in pre-exposed mature-green tomatoes to 500 μL L^−1^ EO-treated after seven days of EO exposure and additional seven days of storage in clean air (as “7 + 7 days” treatment).

The mechanisms underlying the effects of EOs on fruit firmness are unknown. However, it is known that during fruit ripening, cell wall matrices, particularly pectins, are disrupted, and these modifications are thought to be responsible for the decrease in tissue firmness that occurs with ripening [56,57]. In the present study, fruit firmness was maintained in tomatoes-enriched with 50 μL L^−1^ EOs for up to 14 days, compared with higher concentration (500 μL L^−1^) in both red and breaker fruits. The effect of EO was even persisted in fruits pre-exposed to EO (50 μL L^−1^) and stored for an additional seven days in ambient air.

Depending on the species, cultivar, temperature, and climatic and environmental conditions during the growing period, the evolution of total phenolics in fruit during storage could be different [58]. The key contributors to the soluble antioxidant activity in tomato fruit, ascorbic acid, and soluble phenolics increased with storage, resulting in an increase in antioxidant activity in tomato fruit [59]. According to one study, ascorbic acid comprises 28–38% of soluble antioxidant activity, with soluble phenolics accounting for the rest [60]. In our study, increased AA levels were found at two days of EO-treated fruits reflecting the increased DPPH levels found at two and seven days in red tomatoes and at seven days in breaker fruits. Antioxidants help to avoid the build-up of potentially harmful reactive oxygen species (ROS), which are produced as a by-product of cellular metabolism and serve as secondary messengers in hormone signaling [61]. Since tomato fruit is known to be particularly rich in antioxidants [62], such as vitamin C, carotenoids (especially lycopene; [62]), and vitamin A, the antioxidative characteristics of tomato fruit and tomato products are affected by storage procedures, which is a source of worry [63]. Indeed, tomato fruit is an essential nutritional source of several of these compounds, which are vital in the prevention of chronic diseases including heart disease and cancer [64]. The temporary rise in AA content in breaker and red tomatoes (including *β*-carotene in red tomatoes) after two days of EO-enriched atmosphere is noteworthy in this regard. Moreover, EO of 50 μL L^−1^ in red tomatoes kept MDA levels down indicating less stress on the fruits.

During choice testing, panel trials demonstrated a clear preference for EO of 50 μL L^−1^ treated fruits in red tomatoes compared to the untreated fruits, while the opposite was evidenced in breaker tomatoes. Appearance and texture were the main indicators for breaker fruits, while for red tomatoes, not only appearance and texture but also color, aroma, and sweetness were scored to similar levels in low EO-treated fruits and in the control.

## 4. Materials and Methods

### 4.1. Plant Material and Experimental Design

Tomato fruit (*Solanum lycopersicum* L. cv. Dafni F1) was collected from a local field Limassol, Cyprus (crop cultivated for six months under commercial conditions and standard cultural practices in a clay loam soil [65], frequently irrigated by drippers according to crop needs, during spring with temperatures ranging from 18 °C to 28 °C). Fruits were collected by the third inflorescences of the plants. At the laboratory, fruits were selected to obtain homogeneous batches based on color, size, ripeness [breaker stage-mature green (two and three ripening stage)—and light red and red (five and six ripening stage)] and free from defect or injury and then were utilized for experimental purposes. To avoid microbial contamination, the fruits were submerged in a diluted chlorine solution for 3 min before being washed four times with distilled water.

Organic essential oils were extracted by hydrodistillation from sage [*Salvia trilova* L. (Lamiaceae)] gathered in a hilly area of Crete, Greece (without any human inputs) (Clevenger apparatus for 3 h). The composition of the EO was analysed by Gas Chromatography-Mass Spectroscopy (GC-MS), and the main (>2.0%) components were: *α*-Pinene (3.1%), Camphene (2.3%), *β*-Pinene (4.1%), Eucalyptol (53.5%), *cis*-Thujone (6.7%), *trans*-Thujone (3.3%) and Camphor (7.9%), as described previously [36].

Breaker and fully ripe tomato fruits were placed in 1 L polystyrene containers with snap-on lids for each treatment. Two tomatoes were placed in each container, resulting in eight containers (biological replications) per treatment for each of the storage periods. Sage EO used in this study (concentrations based on previous research [22]) were 50 μL L^−1^ and 500 μL L^−1^. Aliquot of each EO solution was placed into individual Eppendorf (1.5 mL) tubes, which were subsequently placed inside the plastic containers shortly before the lids were covered. Filter paper dampened with water was inserted in each container to maintain high relative humidity level during storage, as described in Tzortzakis [22]. The EO volatile components were allowed to spontaneously evaporate inside the containers at 20 °C for 2 h. The containers were then moved to a cold room for storage. Tomato fruit exposed to control (ambient air) or EO (sustainable effect—SE) for 2, 7, and 14 days at 11 °C and 90% relatively humidity (RH~90%) in darkness. Following 1-week exposure, a second batch of fruits were transferred to ambient air and stored at 11 °C for an additional one week (memory effect-ME) named as “7 + 7 d” treatment. To summarize, the experimental set up consisted of 3 treatments × 2 ripening stages × 8 replications (2 fruits per replication) × 4 storage periods (plus day 0) with total of 400 fruits used (Figure 1). Sixteen samples of treated and control fruits were taken after 2, 7, and 14 days and 7 + 7 days for immediate analysis for each ripening stage. For day zero measurements, washed fruits (eight containers) with chlorine were used. Containers were aerated every 72 h avoiding air concentration abnormalities. Volatiles exposure did not cause any phytotoxic effect on the tomato fruit.

### 4.2. Decay Evaluation

After 2, 7, and 14 days of storage at 11 °C, the severity of fruit degradation (in individual fruits in each container; total 16 fruits per treatment per storage period) was visually assessed. Tomato fruit showing surface mycelia growth was considered decayed. On a scale of one to five, the degree of infection on fruit was rated: 1-clean, no infection, 2-trace infection, 3-slight infection, 4-moderate infection, and 5-severe infection. Rots were distinguished by tomato tissue subculture onto Potato Dextrose Agar (PDA) media as described previously [66].

### 4.3. Respiration Rate and Ethylene Emission

The carbon dioxide (CO_2_) and ethylene production were measured by placing each tomato in a 1 L glass jar hermetically sealed with a rubber stopper for 1 h at ambient room temperature. Fruits were weighed and volume was measured. Additionally, CO_2_ and ethylene of room air were tested and subtracted from the measurements, by equipment zeroing, prior to and during experimentation. For respiration rate determination, the holder atmosphere was sucked by a dual gas analyzer (International Control Analyser Ltd., UK) for 30 s. Results were the mean of two determinations for each jar (eight jars per treatment and storage period; *n* = 8) and expressed as milliliter of CO_2_ per kilogram per hour. Ethylene was quantified by using an ethylene analyzer (ICA 56 Analyser, International Control Analyser Ltd., UK) whereas container air sample was sucked for 30 s. Results were the mean of two determinations for each jar and expressed as microliter of ethylene per kilogram per hour (eight jars per treatment and storage period; *n* = 8). CO_2_ and ethylene evolution were calculated according to the following Equation: rate of evolution = M × (V_1_ − V_2_) × (1/w) × (1/t); where, M represents the measurement; V_1_, V_2_ represent jar and fruit volume (mL), respectively; w represents fruit weight (g); and t represents incubation time (h).

### 4.4. Weight Loss, Colour and Fruit Firmness

Individual tomato weights were measured on the day of harvesting (day 0) and after the different sampling dates. Weight loss was calculated for each fruit (*n* = 8) per treatment and storage time as follows: weight loss % = 100 (*W*_o_ − *W*_f_)/*W*_o_, with *W*_o_ being the initial weight and *W*_f_ the final weight of the fruit.

Color was determined using the Hunter Lab System and a Minolta colorimeter model CR400 (Konica Minolta, Osaka, Japan). Following the recording of individual L*, a*, and b* parameters, and chroma value (C) was calculated by the following equations C = (a^*^^2^ + b^*^^2^)^1/2^ as described previously [24]. Results were the mean of determinations made on four points for each fruit (*n* = 8) along the equatorial axis, for each treatment and storage time.

Fruit firmness was measured at two points on the shoulder of each tomato fruit (1 cm^2^ of skin removed), respectively for each treatment by applying a plunger of 8 mm in diameter, using a texturometer FT 011 (TR Scientific Instruments, Forli, Italy). The amount of force (in Newtons; N) required to break the radial pericarp (i.e., surface) of each tomato (*n* = 8) was recorded at ambient (21–23 °C) temperature for each treatment and storage time.

### 4.5. Soluble Solids, Titratable Acidity, Ripening Index, Ascorbic Acid and Carotenoids

Total soluble solids concentration was determined in triplicate from the juice obtained from two pooled tomatoes for each replication (*n* = 8) with a temperature-compensated digital refractometer (model Atago PR-101, Atago Co. Ltd., Tokyo, Japan) at 20 °C, and results were expressed in percentage (%). The titratable acidity was measured via potentiometric titration (Mettler Toledo DL22, Columbus, OH, USA) of 5 mL juice diluted to 50 mL with distilled water using 0.1 N NaOH up to pH 8.1. The results were expressed as percentage of citric acid. The ratio of TSS/TA was used to evaluate the sweetness/ripening index of the fruit.

Ascorbic acid (being the major part in Vitamin C) in eight independent pools of tomato juice was determined by the 2,6-Dichloroindophenol titrimetric method [67]. An aliquot of 5 mL of pooled tomato juice was diluted with 5 mL of water and was titrated by the dye solution until the color changed. Data were expressed as mg of ascorbic acid per gram of fresh weight.

Carotenoids (lycopene and *β*-carotene) were determined according to the Nagata and Yamashita [68] method following modification [69]. Eight individual samples (each sample pooled of two fruits) were examined per treatment and storage period. Thus, 1 g of blended tomatoes were placed in 50 mL falcons and stored in −20 °C till analysis (within 48 h). A volume of 16 mL of acetone:hexane 4:6 (*v:v*) were added to each sample, the samples were shaken vigorously and the two phases were separated automatically. An aliquot was taken from the upper solution for measurement of optical density at 663, 645, 505, and 453 nm in a spectrophotometer, using a reference acetone:hexane (4:6) ratio. Lycopene and *β*-carotene contents were calculated according to the Nagata and Yamashita [68] equations:

Lycopene (mg 100 mL^−1^ of extract) = −0.0458 × A663 + 0.204 × A645 + 0.372 × A505 − 0.0806 × A453.

β-carotene (mg 100 mL^−1^ of extract) = 0.216 × A663 − 1.22 × A645 − 0.304 × A505 + 0.452 × A453.

Results were expressed as nmol per gram of fresh weight.

### 4.6. Total Phenols and Antioxidant Activity

Eight individual samples (each sample pooled of two fruits) were examined per treatment and storage period. Samples of 5 g were milled in an Ultraturrax (T25 digital ultra-turrax, IKA, Germany) with 10 mL methanol (50% *v/v*) for 30 s, and polyphenol extraction was assisted with ultrasound (Ultrasonic cleaning baths-150, Raypa, Spain) for 5 min. The slurry was centrifuged for 30 min on 5000× *g* at 4 °C (Sigma 3–18 K, Sigma Laboratory Centrifuge, Germany). The supernatant was transferred to a 15 mL falcon tube, and was stored at 4 °C until analysis (within 48 h) for evaluation of total phenolic content and total antioxidant activity.

The total phenols content of the methanolic extracts was determined by using Folin–Ciocalteu reagent (Merck), according to the procedure described by Tzortzakis et al. [70]. Briefly, 125 μL of plant extract was mixed with 125 μL of Folin reagent. The mixture was shaken, before addition of 1.25 mL of 7% Na_2_CO_3_, adjusting with distilled water to a final volume of 3 mL, and thorough mixing. After incubation in the dark for 90 min, the absorbance at 755 nm was measured versus the prepared blank. Total phenolic content was expressed as μmol of gallic acid equivalents (GAE) per gram of fresh weight, through a calibration curve with gallic acid. All samples were analyzed in triplicate.

A sample of 3 mL of freshly prepared ferric-reducing antioxidant power solution (0.3 mol L^−1^ acetate buffer, pH 3.6), containing 10 mmol L^−1^ TPTZ (Tripyridil-s-triazine) and 40 mmol L^−1^ FeCl_3_·10H_2_O and 20 μL of extract (50 mg mL^−1^) were incubated at 37 °C for 4 min and the absorbance was measured at 593 nm. The absorbance change was converted into a FRAP value, by relating the change of absorbance at 593 nm of the test sample to that of the standard solution of trolox ((±)-6-Hydroxy-2,5,7,8-tetramethylchromane-2-carboxylic acid). Standard curve was prepared using different concentrations of trolox, and the results were expressed as mg trolox per gram of fresh weight [69]. All samples were analysed in triplicate.

Radical-scavenging activity was determined according to Wojdylo et al. [71] with some modifications. The 2,2-diphenyl-1-picrylhydrazyl radical scavenging activity of the plant extracts was measured from the bleaching of the purple-colored 0.3 mM solution of DPPH. One milliliter of the DPPH solution in ethanol, 1.98 mL (50% *v/v*) methanol and 0.02 mL of plant extract were mixed. After shaking, the mixture was incubated at room temperature in the dark for 30 min, and then the absorbance was measured at 517 nm. The results were expressed in mg trolox per gram of fresh weight. All samples were analyzed in triplicate.

### 4.7. Plant Stress Indicators

Cell damage index of lipid peroxidation in leaves was assessed in terms of malondialdehyde content, which was determined by the thiobarbituric acid reaction [72]. Hydrogen peroxide content was measured according to the method of Loreto and Velikova [73]. The results were expressed as nmol MDA or μmol H_2_O_2_ per g FW. Four replicates were analyzed for each treatment and sampling date.

### 4.8. Sensory Evaluation

For the sensory evaluation, 14 panelists of similar ratio of males and females (aged from 22 to 44 years old) were employed to assess fruit of the two ripening stages and subject to storage for 14 days in ambient air or EO-enriched air (50 μL L^−1^ or 500 μL L^−1^). All panelists had at least some training in the sensory evaluation of tomato fruit. To ensure representative results, the panel was initially asked to assess treatment preferences, with each panelist being given more than one fruit from each sample. Panelists were subsequently challenged with fresh fruit from each treatment and asked to rate appearance, colour, aroma, sweetness, texture, and marketability using scales (values of acceptance) with anchor points 1: ‘Poor/unsweet/soft’ and 5: ‘excellent/very sweet/firm’. Scales were converted to percentage values. Individual panelists were given two sets of fruit (representing the two stages of ripening) and each set had three plates (one for each treatment) containing three whole tomato fruits and three halved tomato fruits for sensory analysis, all tests being conducted under the same conditions and with no time limit. To avoid intermixing of panel members, panel testing was conducted in isolation in booths in the same room.

### 4.9. Statistical Analysis

The data were checked for normality before being subjected to an analysis of variance (ANOVA). The time of storage and the treatments were the sources of variation. Following one-way ANOVA, significant differences between mean values were detected using Tukey’s HSD test (*p* = 0.05). SPSS was used to conduct statistical analysis (SPSS Inc., Chicago, IL, USA).

## 5. Conclusions

The current study emphasizes the possibility of employing natural volatiles obtained from sage essential oils to preserve tomato fruit during storage and/or transit at 11 °C and high RH levels of 90%. In breaker tomatoes, EO-enrichment (sustained effect) retained fruit firmness, respiration rates, and ethylene emission in low EO levels (50 μL L^−1^), while fruit metabolism was sped up in high EO levels of 500 μL L^−1^, with decreased firmness and increased rates of respiration and ethylene and effects on antioxidant capacity. The effects were more pronounced during the storage period of 14 days, in comparison with fruit subject to traditional storage/transit practice. In red fruits, the EOs impacts were evidenced earlier (at two and seven days of storage) with increased rates of respiration and ethylene, increased TSS and *β*-carotene, and decreased lycopene content. Considering the pre-exposed fruits to EOs, quality attributes were more affected in mature green fruits and to a lesser extent in red fruits. Furthermore, based on appearance, color, and texture evaluations, taste panel trials demonstrated an overwhelming preference for EO-treated red fruit during choice testing. Additional investigation is needed to encapsulate the EOs and to examine the application of EOs mixtures, based on their active ingredients, for the preservation of tomato fruits. The use of natural products to preserve fresh commodities should be researched further to determine the best application conditions (i.e., method, duration, and concentration) for each commodity in each case.

## Figures and Tables

**Table 1:** Effect of sage essential oil (EO) of tomato fruit decay at breaker and red ripening stage, exposed to ambient air (control) or EO (50 or 500 μL L^−1^) for 14 days (Sustain effect—S) or up to 7 days and then transferred to ambient air for an additional 7 days (memory effect—M). Fruit were maintained throughout at 11 °C and 90% RH. The degree of infection on fruit was rated using a scale of 1 to 5 (1-clean, no infection; 2-trace infection; 3-slight infection; 4-moderate infection; 5-severe infection).

		Fruit Decay					
	Treatments	Breaker Stage				Red Stage	
		0 Days	7 Days	14 Days	0 Days	7 Days	14 Days
S/M	Control	1.00 ± 0.00 *	1.17 ± 0.12 a	2.05 ± 0.15 a	1.00 ± 0.00 *	1.16 ± 0.10 a	2.75 ± 0.22 a
S	EO-50 μL L^−1^		1.03 ± 0.04 a	1.10 ± 0.15 b		1.06 ± 0.07 a	1.25 ± 0.10 b
S	EO-500 μL L^−1^		1.00 ± 0.00 a	1.00 ± 0.00 b		1.00 ± 0.00 a	1.00 ± 0.00 b
M	EO-50 μL L^−1^			1.15 ± 0.10 b			1.40 ± 0.20 b
M	EO-500 μL L^−1^			1.30 ± 0.25 b			1.95 ± 0.35 ab

Values represent the mean (±SE) of evaluation made on eight independent fruit per treatment per storage period. Values followed by the same letter in each column do not differ significantly (*p* < 0.05). Symbols of * indicating significance among controls through storage period.

**Table 2:** Quantitative analysis of the impacts of sage essential oil (EO) on sensory attributes of tomato fruit at breaker and red ripening stage, exposed to ambient air (control) or EO (50 or 500 μL L^−1^) for 14 days. Fruits were maintained throughout at 11 °C and 90% RH. Values represent mean (±SE) of assessments made by 14 panelists per treatment.

	Breaker Tomatoes			Red Tomatoes		
	Control	EO-50 μL L^−1^	EO-500 μL L^−1^	Control	EO-50 μL L^−1^	EO-500 μL L^−1^
**Appearance**	61.8 ± 4.6 a	64.4 ± 4.9 a	40.0 ± 4.2 b	71.8 ± 3.4 a	78.8 ± 3.9 a	38.5 ± 4.4 b
**Color**	70.1 ± 5.8 a	52.8 ± 5.4 b	50.1 ± 5.8 b	74.2 ± 4.9 a	79.5 ± 3.3 a	51.4 ± 5.0 b
**Aroma**	70.0 ± 5.7 a	57.0 ± 4.5 b	24.2 ± 2.2 c	68.5 ± 4.1 a	67.1 ± 4.5 a	27.1 ± 2.6 b
**Texture**	65.8 ± 5.7 a	72.7 ± 6.4 a	34.2 ± 3.8 b	65.2 ± 3.8 a	74.0 ± 3.5 a	30.0 ± 3.4 b
**Sweetness**	45.7 ± 3.8 a	38.5 ± 3.9 ab	30.0 ± 4.1 b	67.1 ± 5.7 a	59.2 ± 3.8 a	32.8 ± 4.5 b
**Satisfaction**	61.4 ± 3.9 a	49.7 ± 5.3 b	22.8 ± 1.9 c	68.5 ± 4.0 a	55.7 ± 5.2 b	21.4 ± 1.4 c
**Marketability**	67.1 ± 5.7 a	54.7 ± 5.8 b	21.4 ± 1.4 c	80.0 ± 5.5 a	64.2 ± 7.6 b	20.0 ± 0.0 c

In each row, mean values in percentage followed by the same small (breaker stage) are not significantly different, *p ≤* 0.05.

## Supplementary Materials

The following are available online at https://www.mdpi.com/article/10.3390/plants10122645/s1. Figure S1: Impacts of sage essential oil (EO) on *L**, *a**, *b** and chroma values in tomato fruit at breaker and red ripening stage, exposed to ambient air (control) or EO (50 or 500 μL L^−1^).
