# Supplementary material for: Vapour Application of Sage Essential Oil Maintain Tomato Fruit Quality in Breaker and Red Ripening Stages
Source: Plants (Basel). 2021 Dec 1;10(12):2645. doi: 10.3390/plants10122645 (PMC8703985; doi:10.3390/plants10122645)
Supplement: Supplementary file 1 [file plants-10-02645-s001.zip › plants-1487121-supplementary.pdf]

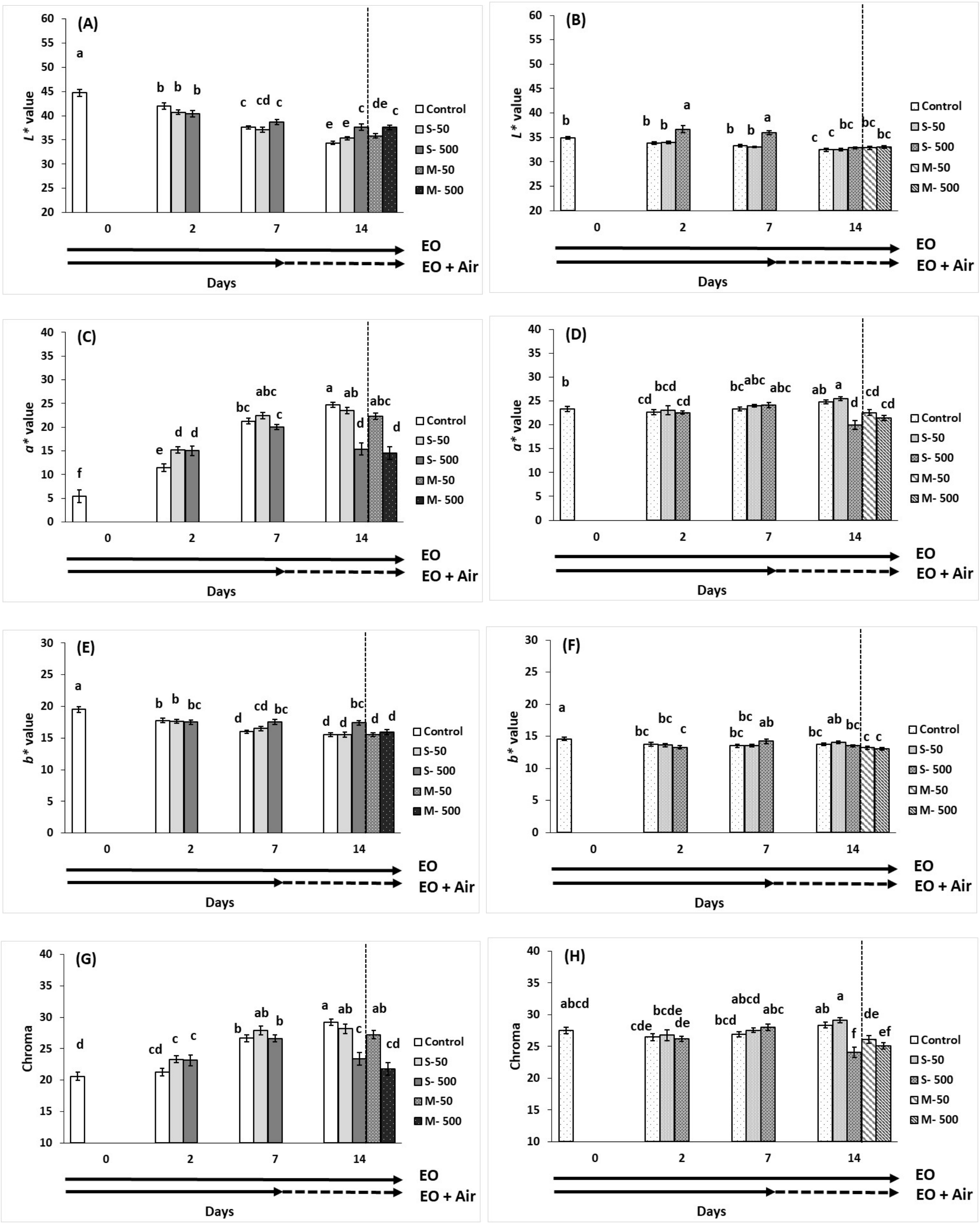

Figure S1. Impacts of sage essential oil (EO) on *L**, *a**, *b** and chroma values in tomato fruit at breaker (A, C, E, G) and red (B, D, F, h) ripening stage, exposed to ambient air (control) or EO (50 or 500 μL L⁻¹) for 2, 7 and 14 days (sustain effect -S) or up to 7 days and then transferred to ambient air for an additional 7 days (memory effect -M). Fruit were maintained throughout at 11 °C and 90 % RH. Values represent mean (± SE) of measurements made on ten independent fruit per treatment and storage period. Means followed by different Latin letters significantly differ according to Duncan's MRT (p = 0.05).
